# Supplementary material for: Tumor heterogeneity and clonal cooperation influence the immune selection of IFN-γ-signaling mutant cancer cells
Source: Nat Commun. 2020 Jan 30;11:602. doi: 10.1038/s41467-020-14290-4 (PMC6992737; doi:10.1038/s41467-020-14290-4)
Supplement: Supplementary file 1 — Supplementary Information [file 41467_2020_14290_MOESM1_ESM.pdf]

Tumor heterogeneity and clonal cooperation influence the immune selection of IFN-γ-signaling mutant cancer cells

Williams et al.

Supplementary Information

Supplementary Figures 1-11
Supplementary Tables 1-2

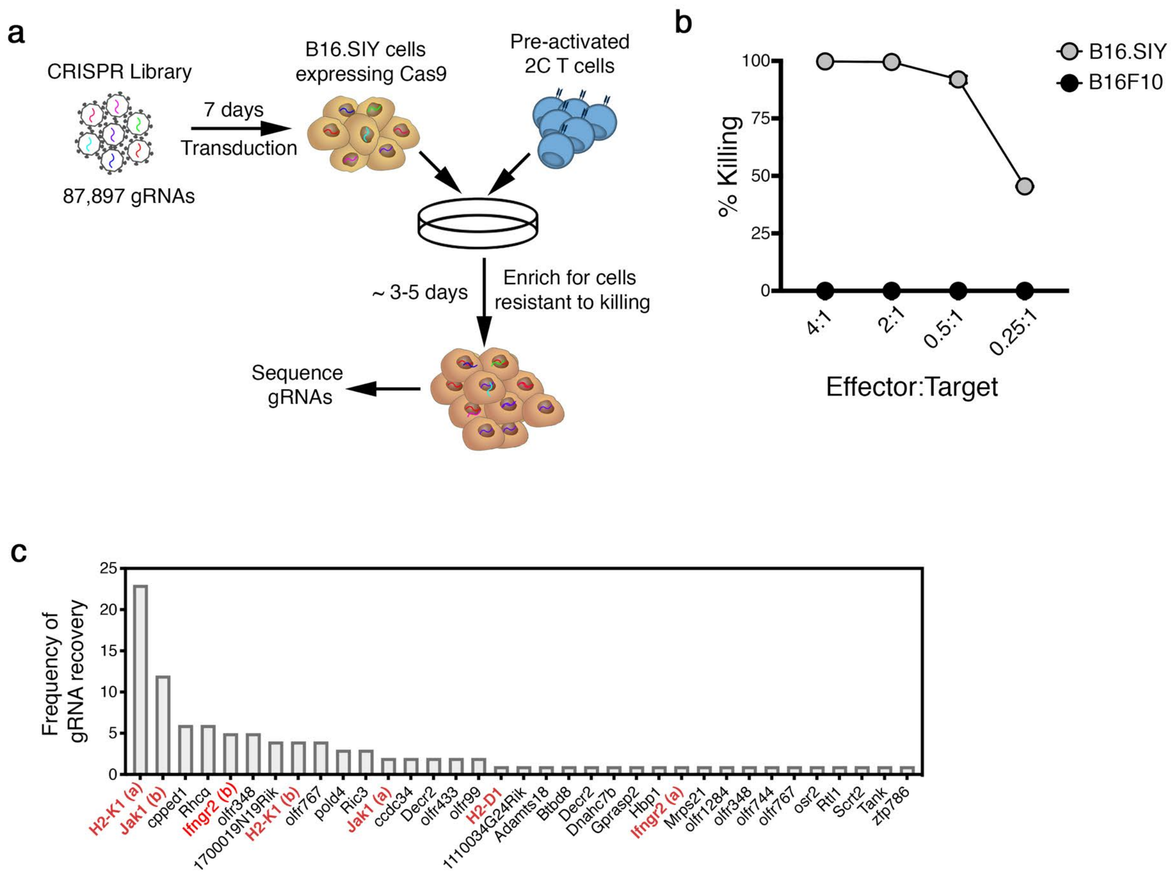

Supplementary Fig. 1. A genome-wide CRISPR/Cas9 screen to identify factors rendering tumor cells resistant to T cell-mediated killing in vitro. **a** Schematic of the genome-wide CRISPR/Cas9 screening approach. A plasmid encoding Cas9 and a blasticidin resistance gene (Cas9-Bsr) was transfected into B16.SIY tumor cells and maintained in blasticidin-containing (15 μg/mL) media. A lentivirus encoding genome-wide sgRNAs with a BFP reporter were transduced into B16.SIY cells. We allowed for CRISPR/Cas9-mutagenesis to occur for 7 days. We then co-cultured pre-primed 2C/Rag2$^{-/-}$ T cells at a 2:1 ratio with tumor cells and allowed for T cell-mediated selection to occur and expansion of resistant cells for 3-5 days. DNA was extracted from remaining tumor cells and the recovered gRNAs were determined by sequencing. **b** Bystander killing in our in vitro CRISPR/Cas9 screen. B16.SIY tumor cells were mixed at a 100:1 ratio with B16F10 tumor cells and submitted to in vitro 2C/Rag2$^{-/-}$ T cell mediated killing. The frequency of dead tumor cells was determined staining cells with a live-dead discriminator dye. **c** Frequency of recovered gRNAs.

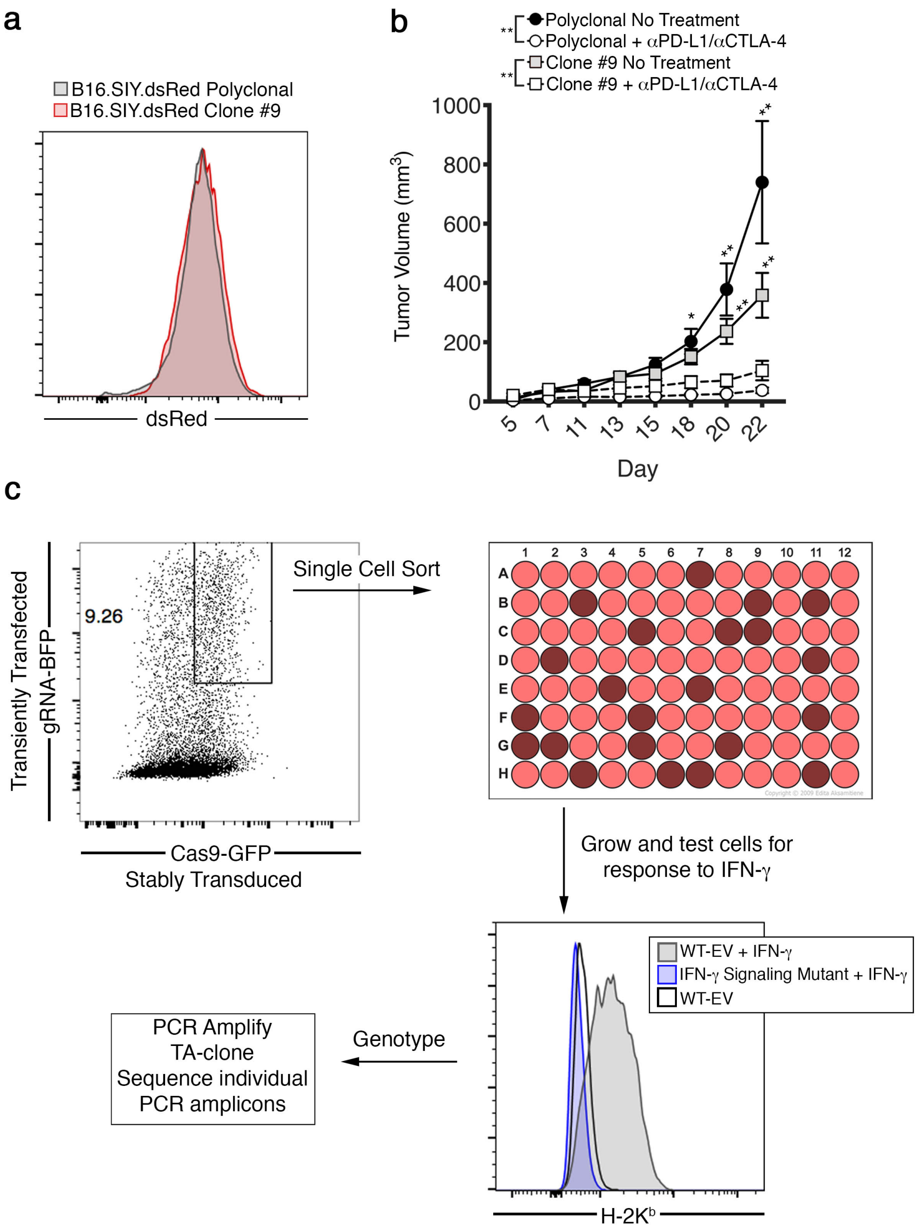

Supplementary Fig. 2. Single-cell cloning technique used to generate IFNγR2- and Jak1-mutant tumor cell lines. **a** SIY.DsRed expression comparing #9 B16.SIY to polyclonal B16.SIY. **b** Tumor outgrowth of polyclonal B16.SIY and #9 B16.SIY tumors in response to anti-PD-L1 + anti-CTLA-4 checkpoint blockade therapy. n=16 mice (polyclonal), n=21 mice (clone #9), n=15 mice (polyclonal + anti-PD-L1/anti-CTLA-4), and n=13 mice (clone #9 + anti-PD-L1/anti-CTLA-4); data are pooled from four independent experiments. **c** Schematic depicting the generation of IFNγR2- and Jak1-mutant tumor cell lines. Detailed methods for each cell line generated are described in the Materials and Methods section. Tumor cells were single-cell sorted based on BFP expression. Single-cell clones were stimulated with IFN-γ for 16 hours and H-2K^b upregulation was measured by flow cytometry. Selected single-cell clones were genotyped by sequencing. Results are expressed as mean ± s.e.m. Statistical significance was determined by a two-way ANOVA Bonferroni post-hoc test (**b**). *$p < 0.05$, **$p < 0.01$, *** $p < 0.001$.

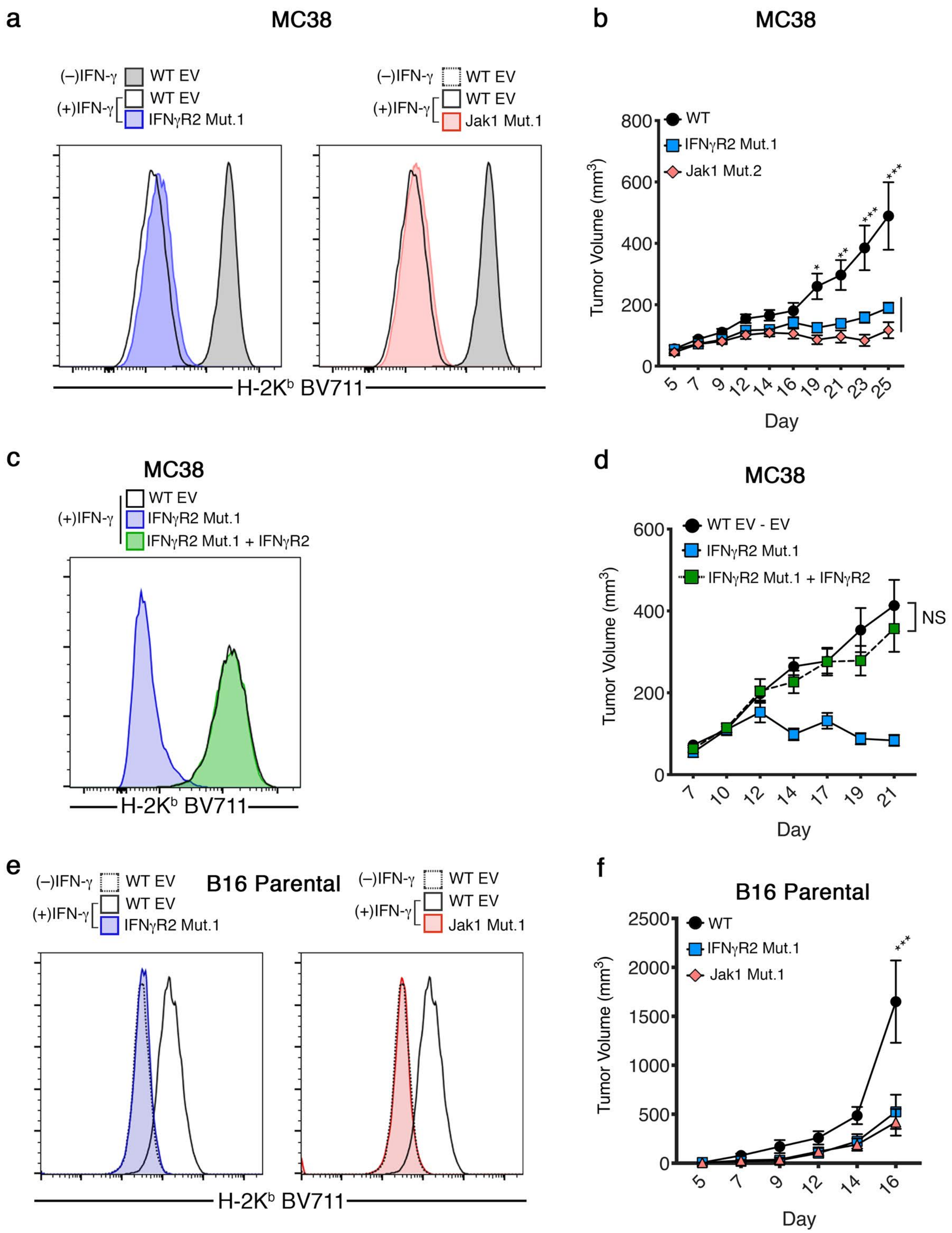

Supplementary Fig. 3. Delayed tumor outgrowth of IFN-γ-insensitive tumors also observed in the B16F10 and MC38 tumor models. **a** Representative histograms of selected MC38 single-cell clones stimulated with IFN-γ and measured for the upregulation of H-2K^b. n=2 stimulations; data is representative of two independent experiments. **b** Tumor outgrowth of IFNγR2- and Jak1-mutant MC38 tumors. n=15 mice (WT and Jak1 Mut.1) and n=10 (IFNγR2 Mut.1); data are pooled from three independent experiments. **c** Representative histogram of restored IFN-γ signaling when IFNγR2 is reintroduced in the MC38 model. **d** Restored progressive tumor growth of MC38 IFNγR2 Mut.1 tumors with re-introduced IFNγR2. n=10 mice; data are pooled from two independent experiments. **e** Representative histograms of selected MC38 single-cell clones stimulated with IFN-γ and measured for the upregulation of H-2K^b. **f** Tumor outgrowth of IFNγR2- and Jak1-mutant B16F10 tumors n=10 mice; data are pooled from two independent experiments. Results are expressed as mean ± s.e.m. Statistical significance was determined by a two-way ANOVA Bonferroni post-hoc test (b, d, f). $^*p < 0.05$, $^{**}p < 0.01$, $^{***}p < 0.001$.

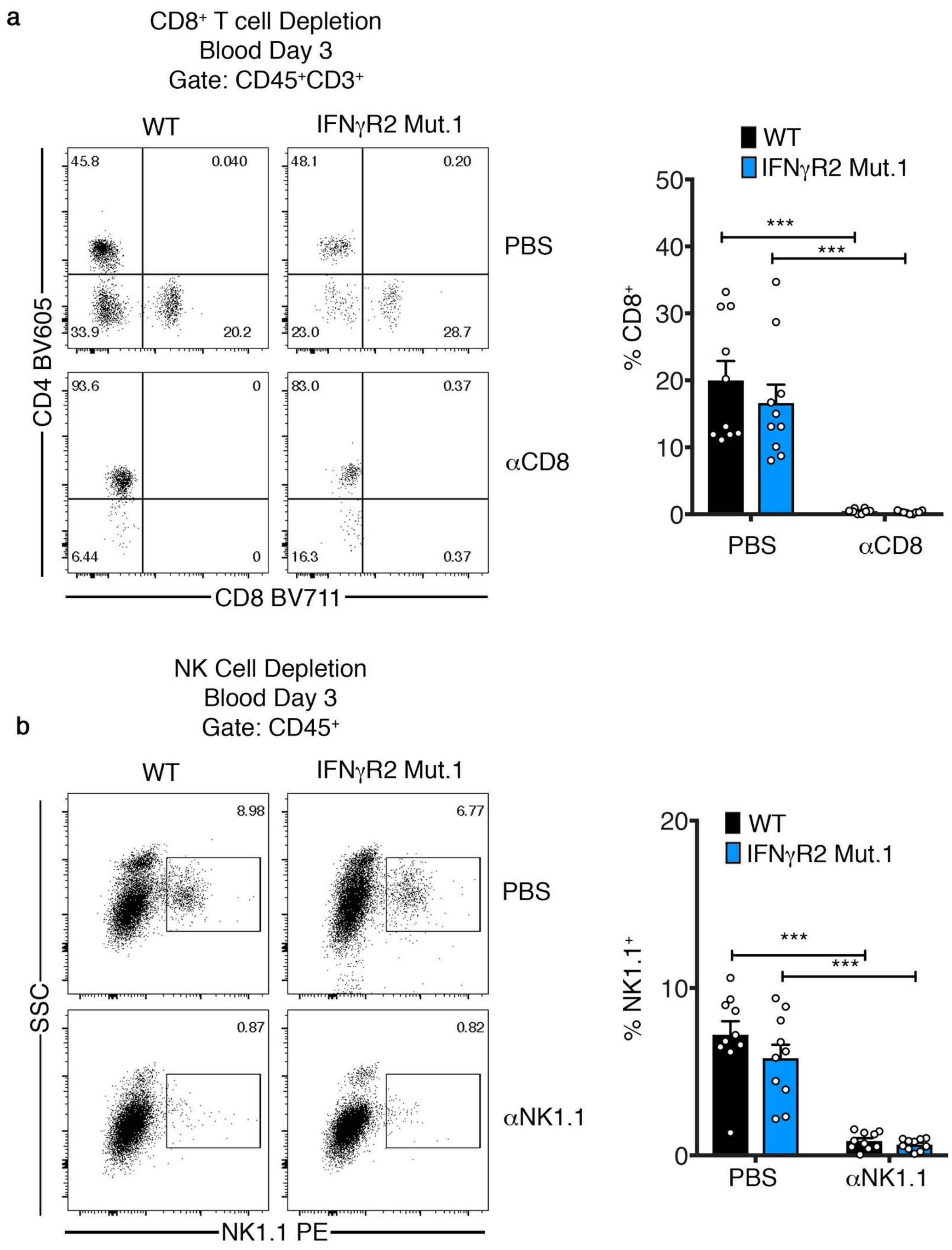

Supplementary Fig. 4. Depletion of CD8⁺ T cells and NK cells. **a** and **b** Representative flow plots and summary of CD8⁺ T cells (**a**) and NK cells (**b**) in the blood 3 days after administration of depleting antibodies compared to the non-treated controls. n=10 (WT and IFNγR2 Mut.1 NT and anti-NK1.1), n=8 (WT + anti-CD8), and n=9 (IFNγR2 + anti-CD8); data are pooled from two independent experiments. Results are expressed as mean ± s.e.m. Statistical significance was determined by a two-way ANOVA Bonferroni post-hoc test (**a, b**). *** $p < 0.001$.

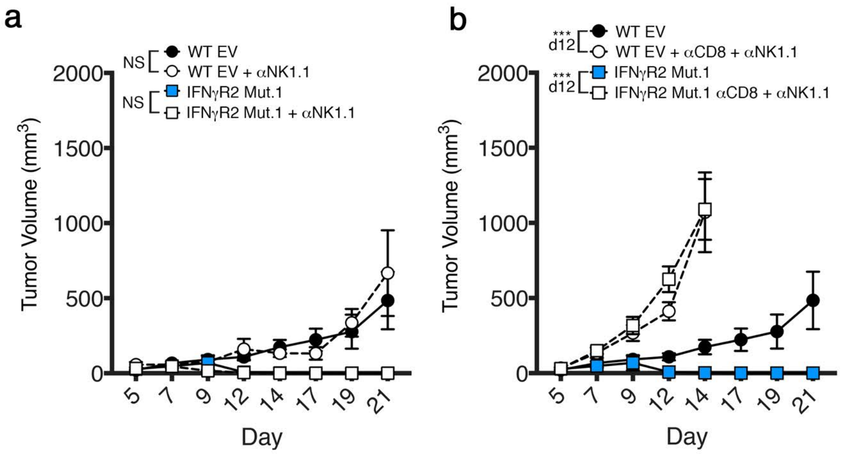

Supplementary Fig. 5. NK cells are not required for spontaneous tumor control of IFN-γ-insensitive tumors. **a** Tumor outgrowth of WT, IFNγR2 Mut.1 tumors in NK cell-depleted mice. Mice received 200 μg of anti-NK1.1 (clone PK136) i.p. 2 days before tumor inoculation and every 7 days throughout the duration of the experiment. **b** Tumor outgrowth of WT, IFNγR2 Mut.1 tumors in $CD8^+$ T cell- and NK cell-depleted mice. As in (**a**), but with combined anti-NK1.1 and anti-CD8α (clone YTS169.4) antibodies. n=10 mice; data are pooled from two independent experiments. Results are expressed as mean ± s.e.m. Statistical significance was determined by a two-way ANOVA Bonferroni post-hoc test (**a**,**b**). *** $p < 0.001$.

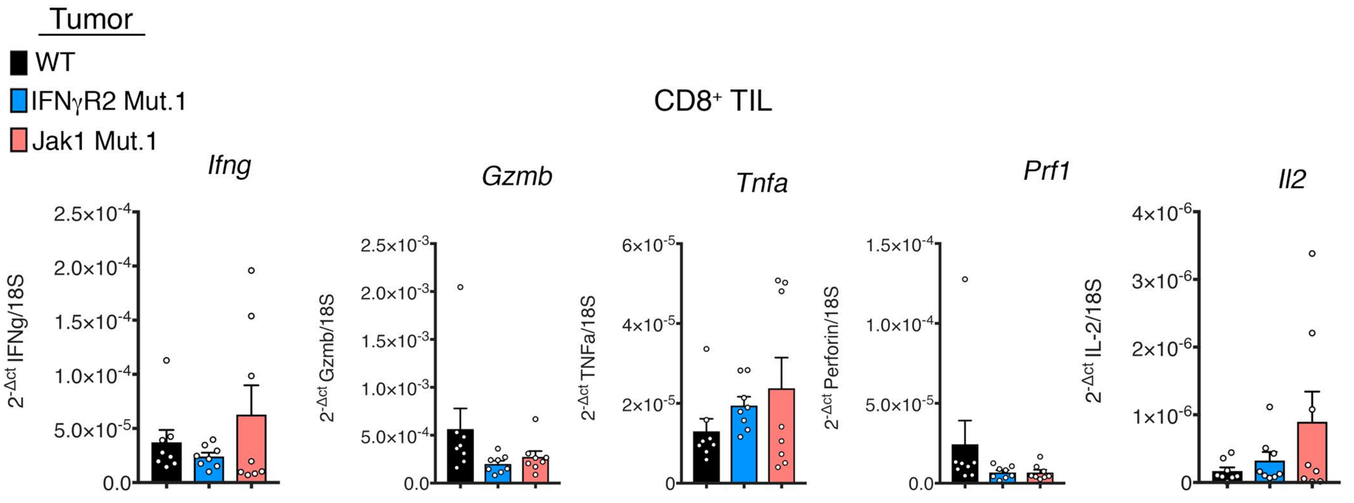

Supplementary Fig. 6. Cytokine production by CD8+ TILs is not increased in the setting of IFN-γ-insensitive tumors. Transcript levels of cytokines in CD8+ TILs. CD8+ TILs were sorted from WT, IFNγR2-, or Jak1-mutant tumor cells on day 7 after tumor engraftment and Ifng, Gzmb, Tnfa, Prf1, and Il2 mRNA was measured by qRT-PCR. n=8 mice; data are pooled from two independent experiments. Results are expressed as mean ± s.e.m. Statistical significance was determined by a Kruskal-Wallis (non-parametric) test.

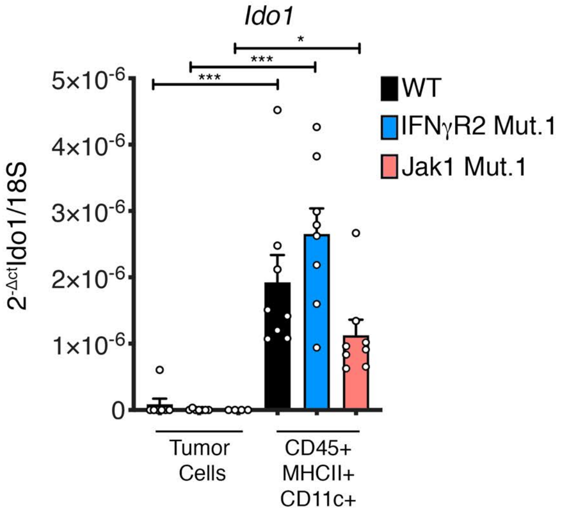

Supplementary Fig. 7. Analysis of Ido1 expression in tumor cells and APCs from the tumor. On day 7 after tumor engraftment Ido expression is predominantly found in host APCs. Transcript levels of Ido in tumor cells and host APCs isolated on day 7 after tumor engraftment. n=7 mice (tumor cells) and n=8 mice (CD11c^{+} cells); data are pooled from two independent experiments. Results are expressed as mean ± s.e.m. Statistical significance was determined by a Kruskal-Wallis (non-parametric) test.

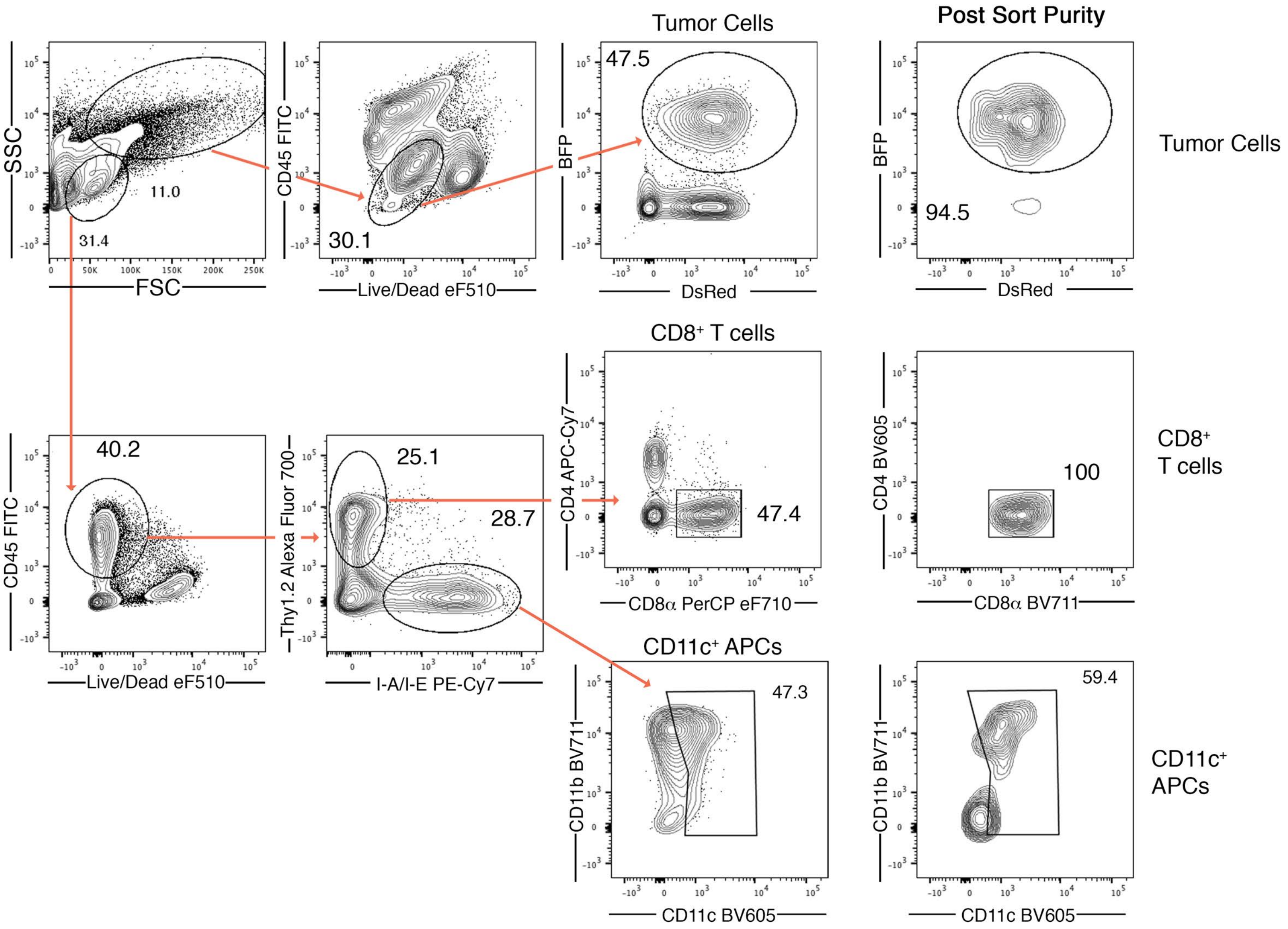

Supplementary Fig. 8. Gating strategy for sorting tumor cells, T cells, and DCs from tumors. This gating strategy was used in Fig. 5, Fig. 6c and d, Supplementary Figure 6, and Supplementary Figure 7.

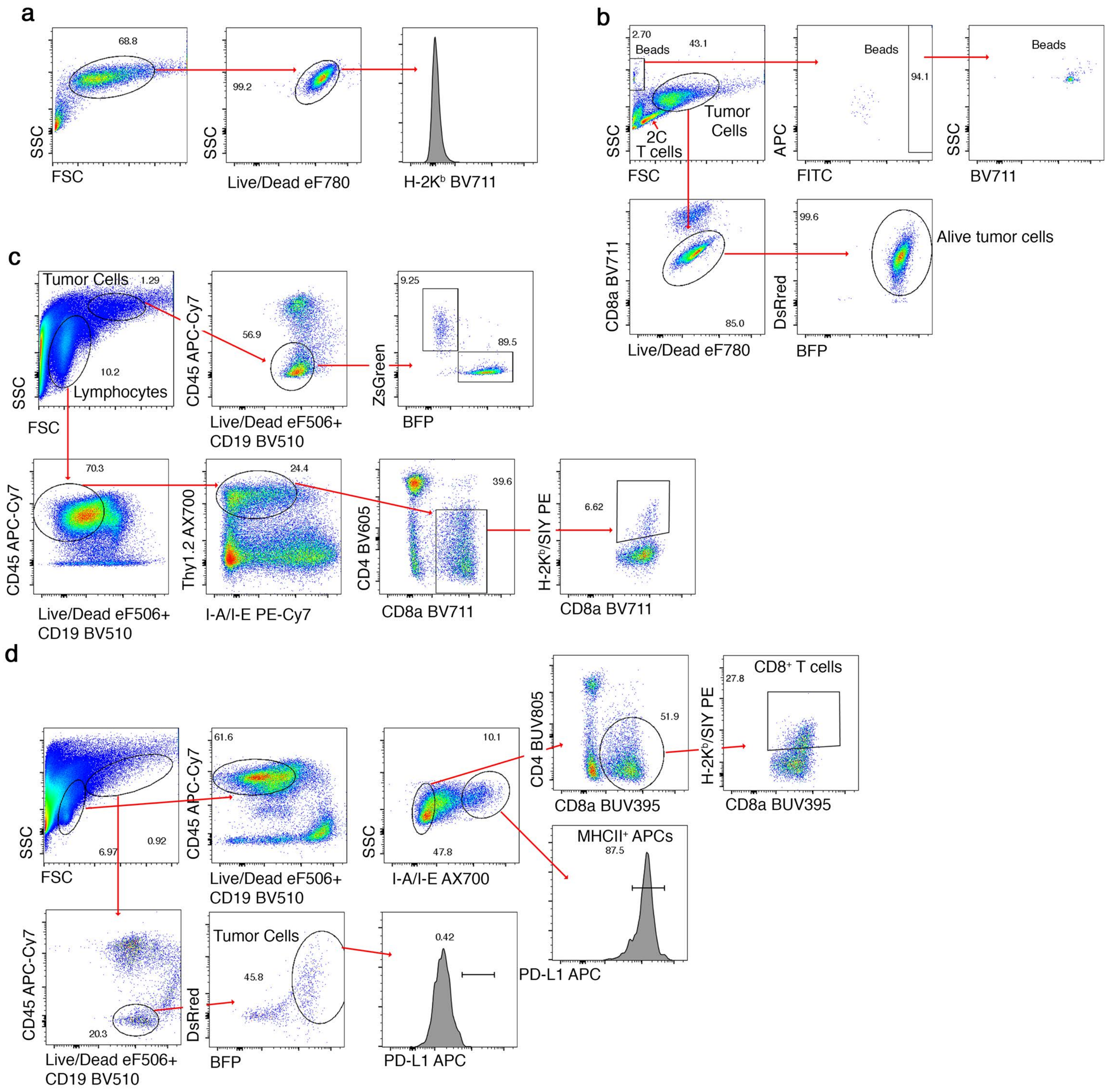

Supplementary Fig. 9. Gating strategy for flow cytometric analyses. **a** Gating strategy for tumor cell stimulations with IFN-γ presented in Fig. 1a, Fig. 2b and c, Fig. 3a (but stained for PD-L1 instead of H-2K^b). **b** Gating strategy for enumerating tumor cells by flow cytometry presented in Fig. 1c. **c** Gating strategy for measuring H-2K^b/SIY$^+$ CD8$^+$ TILs and in some experiments for also measuring the composition of mixed tumors presented in Fig. 3b-d, Fig. 7 b-f and Fig. 8a-f. **d** Gating strategy for measuring PD-L1 expression in vivo presented in Fig. 6a and b.

a IFNγR2 gBlock

GCCGCCACAAGATCTCTCGAGCGGCCGCTAGCAACTGAATTCCGCGGCATAACTTCGTATAATGTATGCTATACGAAGTTATCGATGGCCATGCGGCCTTTGCCACTGTG
GCTGCCGTCGCTGCTGCTCTGTGGGCTCGGTGCCGCGCCGTCCTCGCCAGACTCGTTTTCCCAGCTTGCGGCCCCTCTGAACCCAAGGCTTCACCTGTACAATGATGAGC
AGATTCTAACTTGGGAGCCGTCACCTTCCAGCAATGACCCAAGACCAGTGGTCTACCAGGTGGAATATAGCTTCATAGATGGCTCTTGGCATAGGTTGCTGGAGCCGAAC
TGTACGGACATCACAGAGACAAAGTGTGACTTAACAGGAGGCGGGCGCTTGAAACTTTTCCCACACCCATTCACAGTCTTCCTGCGGGTGCGAGCCAAGCGAGGGAACCT
CACTTCCAAGTGGGTGGGGCTGGAGCCATTTCAACACTATGAGAATGTTACTGTTGGACCTCCCAAGAATATATCAGTCACGCCAGGAAAAGGTTCCCTCGTCATACACT
TCTCCCCTCCGTTTGACGTTTTTCATGGAGCAACTTTTCAGTATCTTGTCCACTACTGGGAAAAGTCAGAAACCCAACAGGAACAGGTTGAAGGCCCTTTCAAGAGCAAC
TCCATTGTGCTGGGCAATCTGAAGCCATACAGAGTATATTGTTTACAAACTGAGGCACAACTGATCTTGAAGAACAAGAAGATCCGGCCACATGGGCTCTTGAGCAATGT
ATCCTGTCACGAAACAACAGCTAATGCCTCCGCCAGGCTACAGCAAGTCATCCTGATTCCGTTGGGCATCTTCGCATTGCTGCTCGGCCTGACGGGCGCCTGCTTCACCC
TGTTCCTCAAATACCAAAGCCGAGTGAAATACTGGTTTCAGGCTCCGCCAAACATCCCGGAACAAATCGAAGAGTATCTAAAGGACCCAGACCAATTCATCTTAGAGGTC
TTGGACAAGGACGGTTCACCGAAGGAGGACTCCTGGGACTCCGTGTCAATTATTTCTTCTCCAGAAAAGGAGCGAGATGATGTGCTCCAAACACCGTGAACCATCGATAA
CTTCGTATAATGTATGCTATACGAAGTTATCCGAATTCGGGGCCCATCGGATCCGTCGACAGATCTCACTCGACACG

b Jak1 gBlock

GCTTTGGAAGGTAAAGAAGAAAATCCAGTCTGCTTTCAGGGACACTGCGGCCGCTCGAGCATAACTTCGTATAATGTATGCTATACGAAGTTATGCGCCATGCAGTATCT
AAATATAAAAGAGGACTGCAATGCCATGGCGTTCTGTGCTAAAATGAGGAGCTTCAAGAAGACTGAGGTGAAGCAGGTGGTCCCTGAGCCTGGAGTGGAGGTGACTTTCT
ATCTGTTGGACAGGGAGCCCCTCCGCCTGGGCAGCGGAGAGTATACAGCCGAGGAACTGTGCATCAGGGCCGCCCAGGAGTGCAGTATCTCTCCTCTCTGTCACAACCTC
TTCGCCCTGTACGATGAGAGCACCAAACTCTGGTACGCTCCAAATCGTATAATTACCGTTGATGACAAAACGTCTCTCCGGCTCCACTACCGCATGAGGTTCTACTTTAC
CAACTGGCACGGAACCAATGACAACGAACAGTCTGTATGGCGACATTCTCCAAAGAAGCAGAAAAACGGCTATGAGAAGAAAAGGGTTCCAGAAGCAACCCCACTCCTTG
ATGCCAGTTCACTGGAGTATCTGTTTGCACAGGGACAGTATGATTTGATCAAATGCCTGGCTCCCATTCGGGACCCCAAGACGGAGCAAGACGGACATGATATTGAAAAT
GAGTGCCTGGGCATGGCGGTCCTGGCCATCTCCCACTATGCCATGATGAAGAAGATGCAGTTGCCGGAACTTCCCAAAGACATCAGCTACAAGCGATATATTCCAGAAAC
ATTGAATAAATCCATCAGACAGAGGAACCTTCTTACCAGGATGCGAATAAATAATGTTTTCAAGGATTTCTTGAAGGAATTTAACAACAAGACCATCTGTGACAGCAGTG
TGTCTACACATGACCTGAAGGTGAAATACCTGGCTACCTTGGAAACTTTGACAAAACATTATGGAGCCGAAATATTTGAGACTTCTATGCTACTGATTTCATCAGAAAAT
GAATTGAGTCGATGCCATTCGAATGACAGTGGCAATGTTCTCTATGAGGTCATGGTGACTGGAAATCTCGGTATCCAGTGGCGGCAGAAACCAAATGTTGTTCCTGTTGA
AAAGGAAAAAAATAAACTGAAGCGGAAAAAACTGGAATATAATAAACACAAGAAGGATGATGAGAGAAACAAACTCCGGGAAGAGTGGAACAATTTTTCCTATTTCCCTG
AAATCACCCACATTGTAATAAAGGAGTCTGTGGTCAGCATTAATAAACAGGACAACAAAAACATGGAACTCAAGCTCTCTTCACGAGAGGAAGCCTTGTCCTTTGTGTCC
CTGGTGGATGGCTACTTCCGGCTCACTGCTGATGCCCACCATTACCTCTGTACTGATGTGGCTCCCCCACTGATTGTCCACAATATACAGAACGGCTGCCACGGTCCAAT
CTGCACAGAATATGCCATCAATAAACTGCGGCAGGAAGGGAGTGAAGAGGGGATGTACGTGCTGAGGTGGAGTTGCACCGACTTTGACAACATTCTTATGACTGTCACCT
GCTTTGAAAAGTCTGAGGTATTGGGTGGCCAGAAGCAGTTCAAGAACTTTCAGATTGAGGTACAGAAGGGCCGCTACAGCCTGCATGGCTCTATGGACCACTTTCCCAGC
CTGCGAGACCTCATGAACCACCTCAAGAAGCAGATCCTGCGCACGGACAACATAAGTTTTGTGCTGAAACGATGCTGTCAGCCTAAGCCTCGGGAAATCTCCAATCTGCT
CGTAGCCACTAAGAAAGCCCAGGAGTGGCAGCCTGTCTACTCCATGAGCCAACTAAGTTTTGATCGAATTCTTAAGAAAGATATTATACAAGGTGAGCACCTTGGCAGAG
GCACAAGAACACATATCTATTCTGGGACCCTGCTGGACTACAAGGATGAGGAAGGAATTGCTGAAGAGAAGAAGATAAAAGTGATCCTCAAAGTCCTAGACCCCAGCCAC
CGGGACATCTCTCTGGCCTTCTTTGAGGCTGCTAGCATGATGAGACAGGTTTCCCACAAACATATAGTGTACCTCTACGGCGTGTGTGTCCGAGATGTGGAAAATATCAT
GGTGGAAGAGTTTGTGGAGGGGGGGCCGTTGGATCTCTTCATGCACCGCAAAAGTGATGCGCTTACTACCCCCTGGAAGTTCAAGGTTGCCAAACAACTGGCCAGTGCCC
TGAGTTACTTGGAAGATAAAGACCTGGTTCATGGAAATGTGTGCACTAAAAACCTCCTTCTGGCCCGTGAGGGCATTGACAGTGACATTGGCCCGTTCATCAAGCTTAGT
GACCCTGGCATCCCAGTCTCTGTGCTGACCAGGCAAGAGTGCATAGAGCGAATCCCCTGGATCGCTCCTGAGTGTGTTGAAGACTCCAAGAACCTGAGTGTGGCTGCTGA
CAAGTGGAGTTTTGGTACCACGCTCTGGGAAATCTGCTACAACGGAGAGATTCCTCTCAAAGACAAGACCCTCATTGAGAAAGAGAGGTTTTATGAAAGCCGCTGCAGGC
CTGTGACTCCATCTTGCAAGGAACTAGCCGACCTCATGACTCGCTGCATGAACTATGACCCCAACCAGAGACCCTTCTTCCGAGCCATCATGAGGGACATTAACAAGCTG
GAGGAGCAGAATCCAGACATTGTTTCAGAAAAGCAGCCAACAACAGAGGTGGACCCCACTCACTTTGAAAAGCGGTTCCTGAAGAGGATTCGTGACTTGGGAGAGGGTCA
CTTTGGGAAGGTTGAACTCTGTAGATATGATCCTGAGGGAGACAACACAGGGGAGCAGGTAGCAGTCAAGTCCCTGAAGCCTGAGAGTGGAGGTAACCACATAGCCGATC
TGAAGAAGGAGATAGAGATCTTACGGAACCTCTACCATGAGAACATTGTGAAGTACAAAGGAATCTGCATGGAAGACGGAGGCAATGGTATCAAACTCATCATGGAGTTT
CTGCCTTCGGGAAGCCTAAAGGAGTATCTGCCAAAGAATAAGAACAAAATCAACCTCAAACAGCAACTAAAATATGCCATCCAGATTTGTAAGGGGATGGACTACTTGGG
TTCTCGGCAATACGTTCACCGCGACTTAGCAGCAAGAAATGTCCTTGTTGAGAGTGAGCATCAAGTGAAGATCGGAGACTTTGGTTTAACCAAAGCAATTGAAACCGATA
AGGAGTACTACACAGTCAAGGACGACCGGGACAGCCCAGTGTTCTGGTACGCTCCCGAATGTTTAATCCAGTGTAAGTTTTATATCGCCTCTGATGTCTGGTCTTTTGGA
GTGACACTGCACGAACTGCTCACTTACTGTGACTCAGATTTTAGTCCCATGGCCTTGTTCCTGAAAATGATAGGCCCAACTCATGGCCAGATGACAGTGACACGGCTTGT
GAATACTCTGAAAGAAGGAAAGCGTCTGCCATGTCCACCCAACTGTCCTGATGAGGTTTATCAGCTTATGAGAAAATGCTGGGAGTTCCAACCATCTAACCGCACAACTT
TTCAGAACCTTATTGAAGGATTTGAAGCACTTTTAAAATAAGAAGCATGAATAACTTCGTATAATGTATGCTATACGAAGTTATACGGGCCCAATCGATAAATTCCCATT
GGATCCTATCAAATCCTTCTCTCCCAAGCCATTTAAAAACGTTTTTTAAGTGAAAAGTTTGTATTCTGCCTCTAAAGTTCCTCAACAAATACT

Supplementary Figure 10. gBlock sequences. **a** Sequence for gBlock encoding IFNγR2. **b** Sequence for gBlock encoding Jak1.

Supplemental Figure 11

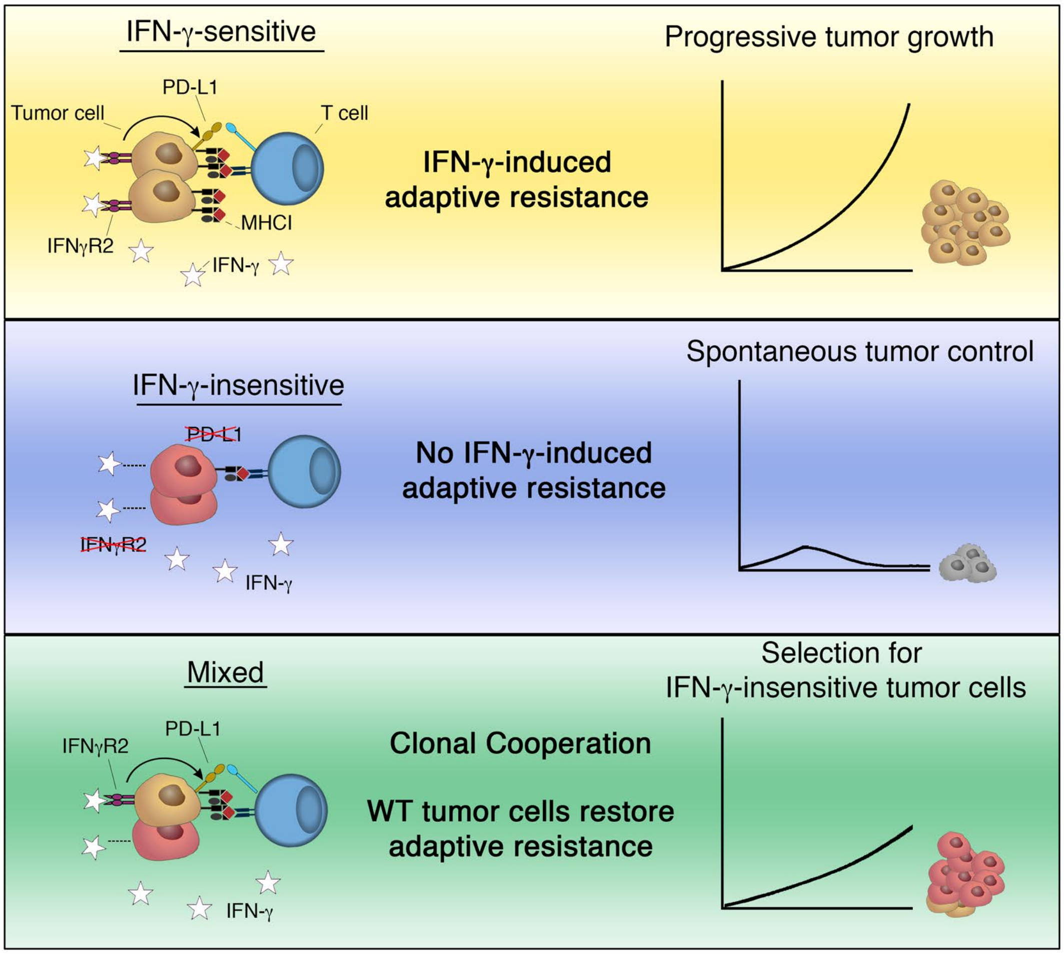

Supplementary Fig. 11. Working model depicting the mechanism behind the spontaneous control of IFN-γ-insensitive mutant tumor cells and selection for these mutants when mixed with WT tumor cells. In the setting of WT tumors IFN-γ-induced adaptive resistant mechanisms, including PD-L1, blunt the initial T cell insult. However, without these adaptive resistance mechanisms IFN-γ-insensitive tumors are better controlled. When mixed with IFN-γ-insensitive tumor cells, WT tumor cells provide the missing adaptive resistance pathways and low MHCI expression on IFN-γ-insensitive tumor cells provides an avenue for selection of these mutants.

Chr	Start	End	Gene	Strand	Target site sequence	PAM	gRNA sequence	clone No.
chr17	33999919	33999942	H2-K1	-	GTACATGGAAGTCGGCTACG	TGG	TACATGGAAGTCGGCTACG	4
chr17	33999458	33999481	H2-K1	-	TACCAGCAGTACGCCTACGA	CGG	ACCAGCAGTACGCCTACGA	23
chr4	101189149	101189172	Jak1	+	CTTGGTGCTCTCATCGTACA	GGG	TTGGTGCTCTCATCGTACA	12
chr4	101189114	101189137	Jak1	+	TCCACAGTGATGATTCGGTT	CGG	CCACAGTGATGATTCGGTT	2
chr16	91559987	91560010	Ifngr2	-	GTCACCGAGATGTTTTTCGG	AGG	TCACCGAGATGTTTTTCGG	5
chr16	91560046	91560069	Ifngr2	+	TCCCTTTGATGTGTTCCACG	GGG	CCCTTTGATGTGTTCCACG	1
chr17	26084055	26084078	Decr2	+	GATGGTGTCAATGTCAACCA	CGG	ATGGTGTCAATGTCAACCA	2
chr7	79601585	79601608	Rhcg	-	TCGGACCTTTTCGCCATGAT	TGG	CGGACCTTTTCGCCATGAT	6
chr16	11887701	11887724	Cpped1	+	GTCACAGTTCCCGGTGGACC	AGG	TCACAGTTCCCGGTGGACC	6
chr10	129079504	129079527	Olfr767	-	CTCTTGGCTAGGTGGGTTGA	TGG	TCTTGGCTAGGTGGGTTGA	1
chr1	174042365	174042388	Olfr433	+	CAGGTACAGCTATGGCTTGC	AGG	AGGTACAGCTATGGCTTGC	2
chr2	36786953	36786976	Olfr348	+	TCTTACTAGTCATTGAATCC	TGG	CTTACTAGTCATTGAATCC	5
chr12	31943842	31943865	Hbp1	+	TCATCACAGGAGTTGTATCC	TGG	CATCACAGGAGTTGTATCC	1
chr2	110018139	110018162	Ccdc34	-	ACTCACGGGCATCTTCGTCC	AGG	CTCACGGGCATCTTCGTCC	2
chr7	109057969	109057992	Ric3	+	GAGTGGTGATGCATCATAGG	TGG	AGTGGTGATGCATCATAGG	3
chr19	4232735	4232758	Pold4	+	TCCTGATGGCAGGTATCACA	AGG	CCTGATGGCAGGTATCACA	3
chr2	61626954	61626977	Tank	+	TTCAACAAAACCTAATTGAC	AGG	TCAACAAAACCTAATTGAC	1
chr19	58789201	58789224	1700019N19Rik	-	GGAGAGAACGCAGAGATACA	AGG	GAGAGAACGCAGAGATACA	4
chr5	107445853	107445876	Btbd8	+	CCCTAACAGGCTTTTAAGGG	AGG	CCTAACAGGCTTTTAAGGG	1
chr17	35263439	35263462	H2-D1	+	GTACATCTCTGTCGGCTATG	TGG	TACATCTCTGTCGGCTATG	1
chr6	47821332	47821355	Zfp786	-	CTCAAAGAGCCGGTCATATA	GGG	TCAAAGAGCCGGTCATATA	1
chr2	111379099	111379122	Olfr1284	+	GCTCTATGTAAGCATCATCG	TGG	CTCTATGTAAGCATCATCG	1
chr12	109594493	109594516	Rtl1	+	TGGAAACATCGCTCGACTCT	GGG	GGAAACATCGCTCGACTCT	1
chr3	95870479	95870502	Mrps21	+	TCGACTCATTTTACCTGTTC	AGG	CGACTCATTTTACCTGTTC	1
chr17	37279882	37279905	Olfr99	-	CCTCATTTTTTCTGTGAATT	TGG	CTCATTTTTTCTGTGAATT	2
chr8	113845074	113845097	Adamts18	-	AGGAAAAGGCGATCGGCACA	CGG	GGAAAAGGCGATCGGCACA	1
chr2	132692105	132692128	1110034G24Rik	-	GATCAGTAGTCGAAGAAGAA	AGG	ATCAGTAGTCGAAGAAGAA	1
chr15	35300622	35300645	Osr2	-	AACGGGGCCGGTCCTTGTGT	AGG	ACGGGGCCGGTCCTTGTGT	1
chr2	152093047	152093070	Scrt2	+	TTTGTCACCCGCAGGTTATG	TGG	TTGTCACCCGCAGGTTATG	1
chr1	46219397	46219420	Dnahc7b	-	GACCTTCCATTAGCTCTCGG	AGG	ACCTTCCATTAGCTCTCGG	1
chrX	135842170	135842193	Gprasp2	-	CCTGGCATCGGTTTTAGGCC	TGG	CTGGCATCGGTTTTAGGCC	1
chr14	50618722	50618745	Olfr744	+	TCTCCCAGGTGTCGTTCTGT	GGG	CTCCCAGGTGTCGTTCTGT	1

Supplementary Table 1. List of recovered gRNAs from the genome-wide CRISPR screen.

ID	Sequence	Use
IFNgR2 gRNA1	TCACCGAGATGTTTTTCGG	guide RNA
IFNgR2 gRNA2	CCCTTTGATGTGTTCCACG	guide RNA
Jak1 gRNA1	CCACAGTGATGATTCGGTT	guide RNA
Jak1 gRNA2	TTGGTGCTCTCATCGTACA	guide RNA
H2kb gRNA1	ACCAGCAGTACGCCTACGA	guide RNA
PDL1 gRNA1	GTAGAAAACATCATTCGCTG	guide RNA
PDL1 gRNA2	GCTTCCCACTCACGGGTTGG	guide RNA
Ifngr2 NF	TCAGAACGGGCATCTGAGTG	Sequencing of targeted region for genotyping
Ifngr2 NR	ATGGTAACAAGACTGAGGGGC	Sequencing of targeted region for genotyping
Jak1 NF	CCAAACACTGCCCCAGGTAA	Sequencing of targeted region for genotyping
Jak2 NR	AGTTGCATCTGCGCTGAGAG	Sequencing of targeted region for genotyping
gRNA F1	AGTTTGGTTAGTACCGGGCC	Used to amplify gRNAs from genomic DNA
gRNA F2	GATCCAAAAAAAGCACCGAC	Used to amplify gRNAs from genomic DNA
hU6F	GAGGGCCTATTTCCCATGATT	Used to sequence the Bbs1 MCS site in the pKLV-
TNFa_Fwd	CTGTAGCCCACGTCGTAGC	qRT-PCR_Used with Probe # 25
TNFa_Rev	TTGAGATCCATGCCGTTG	qRT-PCR_Used with Probe # 25
IFNg_Fwd	GGAGGAACTGGCAAAAGGAT	qRT-PCR_Used with Probe # 21
IFNg_Rev	TTCAAGACTTCAAAGAGTCTGAGG	qRT-PCR_Used with Probe # 21
Gzmb_Fwd	GCTGCTCACTGTGAAGGAAGT	qRT-PCR_Used with Probe # 2
Gzmb_Rev	TGGGGAATGCATTTTACCAT	qRT-PCR_Used with Probe # 2
Perforin_Fwd	GAAGAAGAAACAGCACAAAATGG	qRT-PCR_Used with Probe # 31
Perforin_Rev	GACGTGACGCTCACGGTAG	qRT-PCR_Used with Probe # 31
IL2	Purchased from TaqMan Mm00434256_m1	qRT-PCR
PDL1_Fwd	AAATCGTGGTCCCCAAGC	qRT-PCR_Used with Probe # 64
PDL1_Rev	AATATCCTCATGTTTTGGGAACTATC	qRT-PCR_Used with Probe # 64
IDO1_Fwd	GGGCTTTGCTCTACCACATC	qRT-PCR_Used with Probe # 22
IDO1_Rev	AAGGACCCAGGGGCTGTAT	qRT-PCR_Used with Probe # 22

Supplementary Table 2. Sequences for gRNAs and primer/probe sets for qRT-PCR.
