# Supplementary material for: Tumor heterogeneity and clonal cooperation influence the immune selection of IFN-γ-signaling mutant cancer cells
Source: Nat Commun. 2020 Jan 30;11:602. doi: 10.1038/s41467-020-14290-4 (PMC6992737; doi:10.1038/s41467-020-14290-4)
Supplement: Supplementary file 3 — Reporting Summary [file 41467_2020_14290_MOESM3_ESM.pdf]

nature research

Corresponding author(s): Jason B. Williams
Thomas F. Gajewski

Last updated by author(s): Nov 23, 2019

Reporting Summary

Nature Research wishes to improve the reproducibility of the work that we publish. This form provides structure for consistency and transparency in reporting. For further information on Nature Research policies, see Authors & Referees and the Editorial Policy Checklist .

Statistics

For all statistical analyses, confirm that the following items are present in the figure legend, table legend, main text, or Methods section.

n/a	Confirmed	
☐	☒	The exact sample size (*n*) for each experimental group/condition, given as a discrete number and unit of measurement
☐	☒	A statement on whether measurements were taken from distinct samples or whether the same sample was measured repeatedly
☐	☒	The statistical test(s) used AND whether they are one- or two-sided<br>*Only common tests should be described solely by name; describe more complex techniques in the Methods section.*
☐	☒	A description of all covariates tested
☐	☒	A description of any assumptions or corrections, such as tests of normality and adjustment for multiple comparisons
☐	☒	A full description of the statistical parameters including central tendency (e.g. means) or other basic estimates (e.g. regression coefficient) AND variation (e.g. standard deviation) or associated estimates of uncertainty (e.g. confidence intervals)
☐	☒	For null hypothesis testing, the test statistic (e.g. *F*, *t*, *r*) with confidence intervals, effect sizes, degrees of freedom and *P* value noted<br>*Give P values as exact values whenever suitable.*
☐	☐	For Bayesian analysis, information on the choice of priors and Markov chain Monte Carlo settings
☐	☐	For hierarchical and complex designs, identification of the appropriate level for tests and full reporting of outcomes
☐	☐	Estimates of effect sizes (e.g. Cohen's *d*, Pearson's *r*), indicating how they were calculated

Our web collection on statistics for biologists contains articles on many of the points above.

Software and code

Policy information about availability of computer code

Data collection	No software was used to collect data
Data analysis	Open source software: R<br>Kallisto; https://pachterlab.github.io/kallisto/<br>FastQC: https://cran.r-project.org/web/packages/fastqcr/fastqcr.pdf<br>R script for RNASeq analysis: https://github.com/jaybwilliams1/RNASeqAnalysis_IFNgInsensitiveProject.git

For manuscripts utilizing custom algorithms or software that are central to the research but not yet described in published literature, software must be made available to editors/reviewers. We strongly encourage code deposition in a community repository (e.g. GitHub). See the Nature Research guidelines for submitting code & software for further information.

Data

Policy information about availability of data

All manuscripts must include a data availability statement. This statement should provide the following information, where applicable:
- Accession codes, unique identifiers, or web links for publicly available datasets
- A list of figures that have associated raw data
- A description of any restrictions on data availability

The RNASeq dataset generated during and/or analyzed during the current study are available in the GEO repository, https://www.ncbi.nlm.nih.gov/geo/query/acc.cgi?acc=GSE125698, which will be made available upon publication.
Other datasets (i.e. flow cytometry) generated during and/or analyzed during the current study are available from the corresponding author on reasonable request.

Field-specific reporting

Please select the one below that is the best fit for your research. If you are not sure, read the appropriate sections before making your selection.

[x] Life sciences [ ] Behavioural & social sciences [ ] Ecological, evolutionary & environmental sciences

For a reference copy of the document with all sections, see nature.com/documents/nr-reporting-summary-flat.pdf

Life sciences study design

All studies must disclose on these points even when the disclosure is negative.

Sample size	Each experiment contained 5 mice per group and each experiment was repeated independently at least twice. Given the intensive experimental labor required for mouse models, in certain experiments the sample size is dictated by the personal capacity to throughly and carefully perform an experiment.
Data exclusions	No data was excluded
Replication	All experiments were performed at minimum two times independently.
Randomization	For all experiments using mice, genetically identical mice were purchased from Taconic farms. Mice are shipped in containers containing up to 50 mice. During the delivery process 5 mice are randomly assigned to a cage.
Blinding	For most experiments at least one replicate was blinded. This was performed by having one lab member inoculate mice with the different tumor cell populations and ear tagging mice to identify groups. A different lab member measured tumors sizes.

Reporting for specific materials, systems and methods

We require information from authors about some types of materials, experimental systems and methods used in many studies. Here, indicate whether each material, system or method listed is relevant to your study. If you are not sure if a list item applies to your research, read the appropriate section before selecting a response.

Materials & experimental systems

n/a	Involved in the study	
[ ]	[x]	Antibodies
[ ]	[x]	Eukaryotic cell lines
[ ]	[ ]	Palaeontology
[ ]	[x]	Animals and other organisms
[ ]	[ ]	Human research participants
[ ]	[ ]	Clinical data

Methods

n/a	Involved in the study	
[ ]	[ ]	ChIP-seq
[ ]	[x]	Flow cytometry
[ ]	[ ]	MRI-based neuroimaging

Antibodies

Antibodies used	CD3e (17A2, BioLegend: 100216), Thy1.2 (30-H12, BioLegend: 105320), CD45 (104, BioLegend: 103115), CD8a (53-6.7, BioLegend, 100747), CD4 (RM4-5, BioLegend: 100547), PD-L1 (10F.9G2, BioLegend: 124312), CD19 (6D5, BioLegend: 115545), I-A/I-E (M5/114.15.2, BioLegend: 107630), CD11b (M1/70, BioLegend: 101241), CD11c (N418, BioLegend: 117333), and H-2Kb (AF6-88.5, BD Biosciences: 742862, ThermoFisher: 25-5958-82). For in vivo use: PD-L1 (10F.9G2, BioXCell: BE0101), NK1.1 (PK136, BioXCell: BE0036), CTLA-4 (BE0131, BioXCell: BE0131). For pentamer staining: PE-labeled Pro5 MHC Pentamer H-2Kb SIYRYYGL (ProImmune: F1803-2B - 150 test R-PE).
Validation	All antibodies have been verified by the supplying company.

Eukaryotic cell lines

Policy information about cell lines

Cell line source(s)	Cell lines purchased from ATCC: B16F10, MC38, Hek 293T, and Phoenix cells.
Authentication	Cell line authentication was initially performed by ATCC. Further authentication was performed by microscopy and production of melanin, as B16F10 and MC38 have distinct morphology and B16F10 produce melanin while MC38 cells to not.
Mycoplasma contamination	All cells were routinely tested and tested negative for mycoplasma using Hek-Blue system (InvivoGen)

Commonly misidentified lines (See ICLAC register)	No commonly misidentified cell lines were used in this study.

Palaeontology

Specimen provenance	*Provide provenance information for specimens and describe permits that were obtained for the work (including the name of the issuing authority, the date of issue, and any identifying information).*
Specimen deposition	*Indicate where the specimens have been deposited to permit free access by other researchers.*
Dating methods	*If new dates are provided, describe how they were obtained (e.g. collection, storage, sample pretreatment and measurement), where they were obtained (i.e. lab name), the calibration program and the protocol for quality assurance OR state that no new dates are provided.*

☐ Tick this box to confirm that the raw and calibrated dates are available in the paper or in Supplementary Information.

Animals and other organisms

Policy information about studies involving animals; ARRIVE guidelines recommended for reporting animal research

Laboratory animals	Female C57BL/6NTac mice were purchased from Taconic farms. All mice were of 6-8 weeks of age.
Wild animals	No wild animals were involved in this study.
Field-collected samples	This study did not contain field-collected samples
Ethics oversight	All mouse experiments were in accordance with IACUC-approved protocols.

Note that full information on the approval of the study protocol must also be provided in the manuscript.

Human research participants

Policy information about studies involving human research participants

Population characteristics	*Describe the covariate-relevant population characteristics of the human research participants (e.g. age, gender, genotypic information, past and current diagnosis and treatment categories). If you filled out the behavioural & social sciences study design questions and have nothing to add here, write "See above."*
Recruitment	*Describe how participants were recruited. Outline any potential self-selection bias or other biases that may be present and how these are likely to impact results.*
Ethics oversight	*Identify the organization(s) that approved the study protocol.*

Note that full information on the approval of the study protocol must also be provided in the manuscript.

Clinical data

Policy information about clinical studies

All manuscripts should comply with the ICMJEguidelines for publication of clinical research and a completedCONSORT checklist must be included with all submissions.

Clinical trial registration	*Provide the trial registration number from ClinicalTrials.gov or an equivalent agency.*
Study protocol	*Note where the full trial protocol can be accessed OR if not available, explain why.*
Data collection	*Describe the settings and locales of data collection, noting the time periods of recruitment and data collection.*
Outcomes	*Describe how you pre-defined primary and secondary outcome measures and how you assessed these measures.*

ChIP-seq

Data deposition

☐ Confirm that both raw and final processed data have been deposited in a public database such as GEO.

☐ Confirm that you have deposited or provided access to graph files (e.g. BED files) for the called peaks.

Data access links *May remain private before publication.*	*For "Initial submission" or "Revised version" documents, provide reviewer access links. For your "Final submission" document, provide a link to the deposited data.*
Files in database submission	*Provide a list of all files available in the database submission.*

Genome browser session (e.g. UCSC) | *Provide a link to an anonymized genome browser session for "Initial submission" and "Revised version" documents only, to enable peer review. Write "no longer applicable" for "Final submission" documents.*

Methodology

Replicates | *Describe the experimental replicates, specifying number, type and replicate agreement.*

Sequencing depth | *Describe the sequencing depth for each experiment, providing the total number of reads, uniquely mapped reads, length of reads and whether they were paired- or single-end.*

Antibodies | *Describe the antibodies used for the ChIP-seq experiments; as applicable, provide supplier name, catalog number, clone name, and lot number.*

Peak calling parameters | *Specify the command line program and parameters used for read mapping and peak calling, including the ChIP, control and index files used.*

Data quality | *Describe the methods used to ensure data quality in full detail, including how many peaks are at FDR 5% and above 5-fold enrichment.*

Software | *Describe the software used to collect and analyze the ChIP-seq data. For custom code that has been deposited into a community repository, provide accession details.*

Flow Cytometry

Plots

Confirm that:

- [x] The axis labels state the marker and fluorochrome used (e.g. CD4-FITC).
- [ ] The axis scales are clearly visible. Include numbers along axes only for bottom left plot of group (a 'group' is an analysis of identical markers).
- [ ] All plots are contour plots with outliers or pseudocolor plots.
- [x] A numerical value for number of cells or percentage (with statistics) is provided.

Methodology

Sample preparation | As indicated in the methods section, for isolation of TILs, tumors were dissociated through a 50 um filter in 5-10 mL of PBS. Tumors were washed once with PBS and live cells were purified by Ficoll density gradient. Cells were washed once with PBS containing 2%FBS. For tumor cells, tumors were digested with enzyme mix containing Collagenase (1mg/mL, Sigma: C5138), DNAse (200 ug/mL, Sigma: D5025), and Hyaluronidase (100 ug/mL, Sigma: H6254) for 30 min at 37oC while rotating. Digested tumors were filtered through a 50 um filter in 10 mL of PBS. Cells were washed once with PBS and live cells were isolated by Ficoll density gradient.

Instrument | BD Fortessa 4-15 or BD Fortessa X20 5-18.

Software | BD FACS Diva

Cell population abundance | To determine purity of FACS sorted cells, approximately 1000 cells were first sorted into PBS and re-analyzed on the same FACS cytometer. Samples were then sorted into RLT lysis buffer and immediately frozen to minimizes changes in gene expression.

Gating strategy | For gating strategy and post sort purity see Suppl. Fig. 8.
CD11c+ APCs were gated as follows: FSCmid/SSCmid, CD45(+)Live/Dead(–), Thy1.2(–)MHCII(–), CD8(+)CD4(–)
CD8+ T cells were gated as follows: FSCmid/SSCmid, CD45(+)Live/Dead(–), Thy1.2(+)MHCII(+),CD11c(+)
Tumor cells were gated as follows: FSChi/SSChi, CD45(–)Live/Dead(–), DsRed(+)BFP(+)

- [ ] Tick this box to confirm that a figure exemplifying the gating strategy is provided in the Supplementary Information.

Magnetic resonance imaging

Experimental design

Design type | *Indicate task or resting state; event-related or block design.*

Design specifications | *Specify the number of blocks, trials or experimental units per session and/or subject, and specify the length of each trial or block (if trials are blocked) and interval between trials.*

Behavioral performance measures | *State number and/or type of variables recorded (e.g. correct button press, response time) and what statistics were used to establish that the subjects were performing the task as expected (e.g. mean, range, and/or standard deviation across subjects).*

Acquisition

Imaging type(s) | *Specify: functional, structural, diffusion, perfusion.*

Field strength | *Specify in Tesla*

Sequence & imaging parameters | *Specify the pulse sequence type (gradient echo, spin echo, etc.), imaging type (EPI, spiral, etc.), field of view, matrix size, slice thickness, orientation and TE/TR/flip angle.*

Area of acquisition | *State whether a whole brain scan was used OR define the area of acquisition, describing how the region was determined.*

Diffusion MRI ☐ Used ☐ Not used

Preprocessing

Preprocessing software | *Provide detail on software version and revision number and on specific parameters (model/functions, brain extraction, segmentation, smoothing kernel size, etc.).*

Normalization | *If data were normalized/standardized, describe the approach(es): specify linear or non-linear and define image types used for transformation OR indicate that data were not normalized and explain rationale for lack of normalization.*

Normalization template | *Describe the template used for normalization/transformation, specifying subject space or group standardized space (e.g. original Talairach, MNI305, ICBM152) OR indicate that the data were not normalized.*

Noise and artifact removal | *Describe your procedure(s) for artifact and structured noise removal, specifying motion parameters, tissue signals and physiological signals (heart rate, respiration).*

Volume censoring | *Define your software and/or method and criteria for volume censoring, and state the extent of such censoring.*

Statistical modeling & inference

Model type and settings | *Specify type (mass univariate, multivariate, RSA, predictive, etc.) and describe essential details of the model at the first and second levels (e.g. fixed, random or mixed effects; drift or auto-correlation).*

Effect(s) tested | *Define precise effect in terms of the task or stimulus conditions instead of psychological concepts and indicate whether ANOVA or factorial designs were used.*

Specify type of analysis: ☐ Whole brain ☐ ROI-based ☐ Both

Statistic type for inference
(See Eklund et al. 2016) | *Specify voxel-wise or cluster-wise and report all relevant parameters for cluster-wise methods.*

Correction | *Describe the type of correction and how it is obtained for multiple comparisons (e.g. FWE, FDR, permutation or Monte Carlo).*

Models & analysis

n/a	Involved in the study
☐	☐ Functional and/or effective connectivity
☐	☐ Graph analysis
☐	☐ Multivariate modeling or predictive analysis

Functional and/or effective connectivity | *Report the measures of dependence used and the model details (e.g. Pearson correlation, partial correlation, mutual information).*

Graph analysis | *Report the dependent variable and connectivity measure, specifying weighted graph or binarized graph, subject- or group-level, and the global and/or node summaries used (e.g. clustering coefficient, efficiency, etc.).*

Multivariate modeling and predictive analysis | *Specify independent variables, features extraction and dimension reduction, model, training and evaluation metrics.*
