# Supplementary material for: Tumor heterogeneity and clonal cooperation influence the immune selection of IFN-γ-signaling mutant cancer cells
Source: Nat Commun. 2020 Jan 30;11:602. doi: 10.1038/s41467-020-14290-4 (PMC6992737; doi:10.1038/s41467-020-14290-4)
Supplement: Supplementary file 4 — Description of Additional Supplementary Files [file 41467_2020_14290_MOESM4_ESM.pdf]

Description of Additional Supplementary Files

File Name: Supplementary Data 1
Description: List of differentially expressed genes in tumor cells. Tumor cells were isolated from tumors on day 7 after tumor inoculation. A cutoff fold change > 2 and adjusted p-value < 0.05 was used.
